# Supplementary figures and images for: Lipidome visualisation, comparison, and analysis in a vector space
Source: PLoS Comput Biol. 2025 Apr 15;21(4):e1012892. doi: 10.1371/journal.pcbi.1012892 (PMC12058142; doi:10.1371/journal.pcbi.1012892)

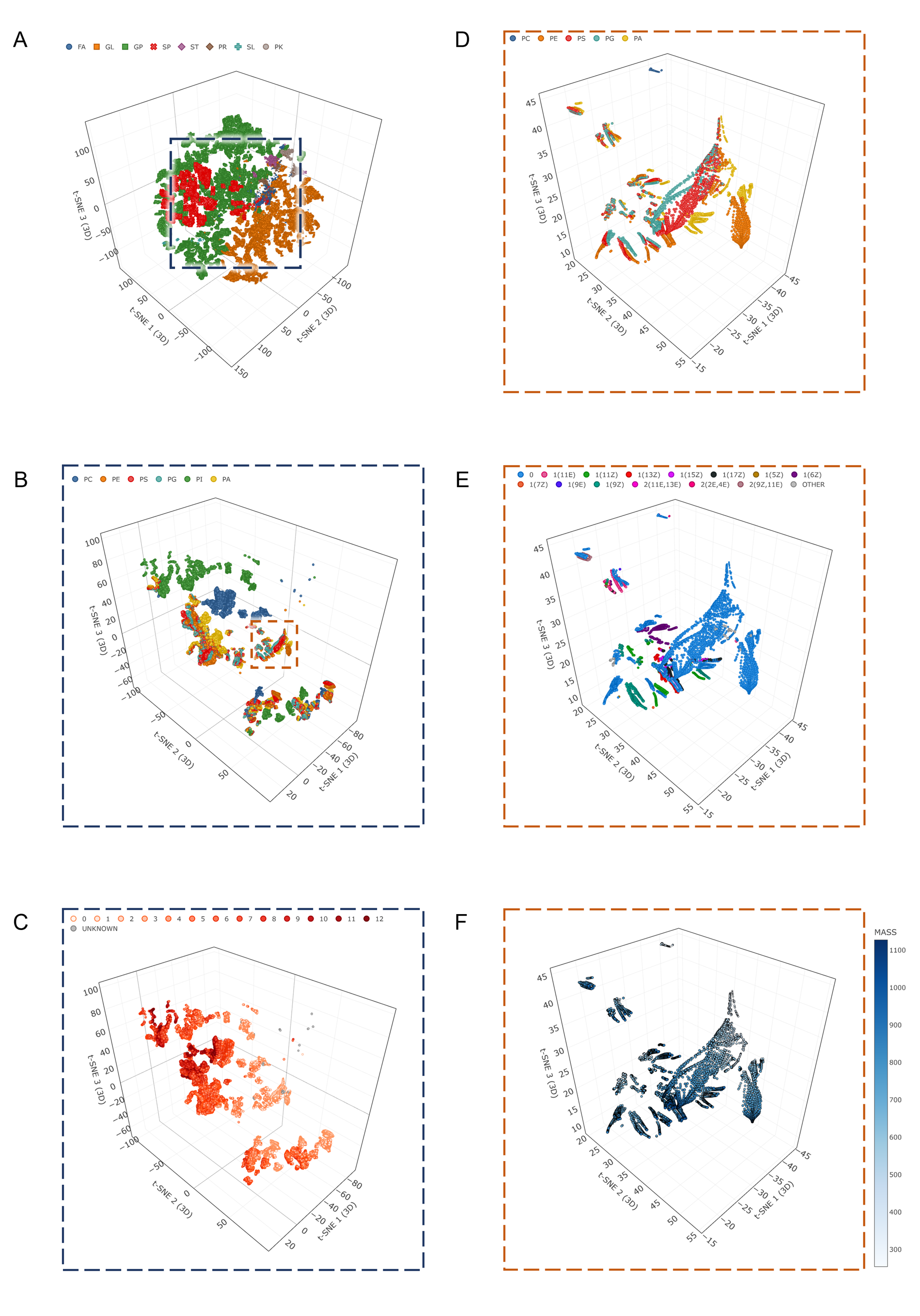

Supplement: S1 Fig — (A) Projection of the entire vector space. Marker colour represents lipid category: Fatty acids (FA), glycerolipids (GL), glycerophospholipids (GP), sphingolipids (SP), sterol lipids (ST), prenol lipids (PR), saccharolipids (SL) and polyketides (PK). (B) Region of the vector space focused on a set of selected glycerophospholipids: Glycerophosphates (PA), glycerophosphocholines (PC), glycerophosphoethanolamines (PE), glycerophosphoglycerols (PG), glycerophosphoinositols (PI) and glycerophosphoserines (PS). Marker colour: Lipid class. (C) Same region as in B. Marker colour: Number of fatty acyl double bonds. (D) Zoomed in region of selected glycerophospholipids. Marker colour: Lipid class. (E) Same region as in D. Marker colour: Double bond profile of the 2-sn fatty acyl. (F) Same region as in D. Marker colour: Molecule mass. See S3 Dataset for interactive HTML. (TIF) [file pcbi.1012892.s001.tif]

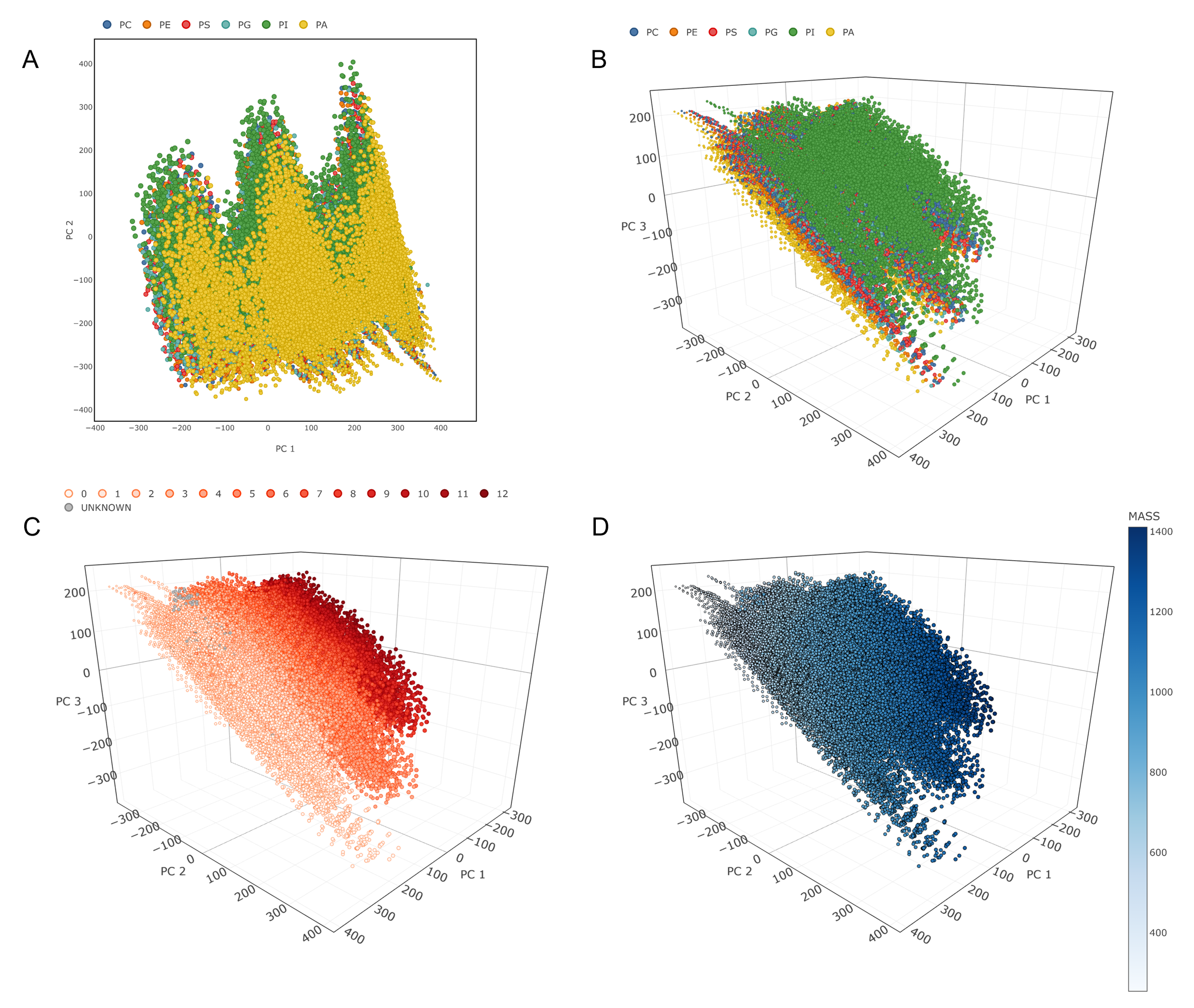

Supplement: S2 Fig — (A) Region of the vector space focused on a set of selected glycerophospholipids: Glycerophosphates (PA), glycerophosphocholines (PC), glycerophosphoethanolamines (PE), glycerophosphoglycerols (PG), glycerophosphoinositols (PI) and glycerophosphoserines (PS). Axes correspond to the first two principal components. (B) Same region and colours as in A. Axes correspond to the first three principal components. (C) Same region and axes as in B. Marker colour: Number of fatty acyl double bonds. (D) Same region and axes as in B. Marker colour: Molecule mass. See S3 Dataset for interactive HTML. (TIF) [file pcbi.1012892.s002.tif]
