# Supplementary material for: Lipidome visualisation, comparison, and analysis in a vector space
Source: PLoS Comput Biol. 2025 Apr 15;21(4):e1012892. doi: 10.1371/journal.pcbi.1012892 (PMC12058142; doi:10.1371/journal.pcbi.1012892)
Supplement: S1 Table — (DOCX) [file pcbi.1012892.s003.docx]

**S1 Table. Word2Vec Embedding Parameters.**

| Word2Vec | | | | | |
| --- | --- | --- | --- | --- | --- |
| Vector size | Context window | Architecture | Optimization | Minimum word frequency | Training iterations |
| 100 | 4 | Skip-gram | Hierarchical softmax | 1 | 10 |
