# Supplementary material for: Lipidome visualisation, comparison, and analysis in a vector space
Source: PLoS Comput Biol. 2025 Apr 15;21(4):e1012892. doi: 10.1371/journal.pcbi.1012892 (PMC12058142; doi:10.1371/journal.pcbi.1012892)
Supplement: S2 Table — (DOCX) [file pcbi.1012892.s004.docx]

**S2 Table. Stochastic Neighbour Embedding Parameters**

| Optimization | Distance metric | Initialization | Perplexity | Early exaggeration | Iterations |
| --- | --- | --- | --- | --- | --- |
| Barnes-Hut | Cosine | Principal component analysis | 150 | 50 | 1000 |
