# Supplementary material for: Lipidome visualisation, comparison, and analysis in a vector space
Source: PLoS Comput Biol. 2025 Apr 15;21(4):e1012892. doi: 10.1371/journal.pcbi.1012892 (PMC12058142; doi:10.1371/journal.pcbi.1012892)
Supplement: Attachment — Submitted filename: review_lipidome_analysis.docx [file pcbi.1012892.s008.docx]

Review for the Manuscript „Lipidome visualisation, comparison, and analysis in a vector space“

The authors present on one hand an embedding of lipids through unsupervised machine learning and on the other hand present a visualisation tool **Lipidome Projector** for the resulting embedded structures.

**Problem definition**: It is clearly defined which specific problem in the field of lipidomic analysis is to be solved by the program. The program contributes to the solution of an existing problem.

**Methodology**: The choice of ML model is clearly described and justified. The data set has been sufficiently described. It is sufficiently large and diverse to provide generalizable results. The training and validation process was well documented.

**Interpretation of the results**: The results were interpreted in a biological context. The program supports the acquisition of knowledge in lipidomics and facilitates data analysis. It is described how the program can be practically applied in lipidomic research (in the analysis of complex lipid patterns).

Potential limitations were also discussed. A more fundamental problem is ambiguities in lipid notation, as well as ambiguities regarding the position, number and exact location of double bonds and hydroxylations in sphingolipids. Another problem is that a user lipidome may contain species that are not included in the training dataset (SwissLipids + LMSD).

**Reproducibility and availability:** The results and methods have been documented in such a way that other researchers can reproduce them. The code is publicly available.

**Contribution:** Lipidome Projector makes it easier to group and classify structurally similar lipids. This enables more efficient identification of patterns and similarities in lipid structure that are more difficult to detect using traditional analytical methods. Similar structures often indicate similar biochemical functions. The program makes it possible to identify functional similarities between lipids more quickly, which supports the study of lipid networks in cells.

**Similar tools:** LipidMatch is another tool that enables comprehensive identification and visualization of lipids based on mass spectrometry data. Although it is mainly used for lipid identification, it also supports the classification of lipids and the visualization of their structural relationships.LIPID MAPS, which provides detailed 2D and 3D structural information on lipids and includes visualization tools for exploring lipid metabolic pathways and interactions. It classifies lipids into major categories and shows their structure-function relationships.

Minor revisions:

- Supporting information „supp-1.csv“ requires a title and a short description what information the table holds for quicker understanding
- It could be important to check for a bias in the substructures available in the databases – due to either underlying biological patterns, differeing detectability in the LC-MS or simply through overrepresentation by resarch interest (e.g. focus on specific diseases) and to add a potential bias into the discussion how generalizable Lipidome Projector could be for edge cases.
- The reproducablility of Lipidome Projector could be improved significantlly if the package verions could either be added in the script, or in the pyproject.toml
- There are two sentences that are a bit misunderstandable:
  - Page 3 – Line 46: „but a list of 10²-104 lipids and quantities“ could be addressed as „lipids and lipid quantities“ or „lipids and their quantities“
  - Page 13 – Line 286-289: „…it is remarkable that moving a hydroxylation along a chain gives a set of points near each other and almost lying on a smooth curve.“ This does not sound grammatically correct.
