# Supplementary material for: Lipidome visualisation, comparison, and analysis in a vector space
Source: PLoS Comput Biol. 2025 Apr 15;21(4):e1012892. doi: 10.1371/journal.pcbi.1012892 (PMC12058142; doi:10.1371/journal.pcbi.1012892)
Supplement: Attachment — Submitted filename: lipidome_analysis_response_to_reviewers.docx [file pcbi.1012892.s009.docx]

Dear Professor Friedberg

Thank you for taking care of the manuscript.

We do consider the points of all three referees, but we begin with two general comments.

1. Two referees mention that t-SNE distorts some relationships as it reduces dimensions.

We have absolutely no disagreement. We have changed the discussion so there is a brutal, critical paragraph making the problems clear. There is an additional principal components figure in the supplementary materials.

2. The display software is an important part of the paper and at some point, one had to have a feature freeze. This is a snapshot. We are grateful for the suggestions, but if something is not broken, the feature will not be added for this publication. Trying to display the properties of 10^5^ points requires some decisions which will not be to every user's taste. Some referee suggestions will be implemented, but will not be in this release.

Reviewer #1:

1. t-SNE is notoriously unstable and can provide structure in data even when there isn't. The structure identified by the authors in, for instance, Fig. 2A has a clear semantic interpretation, which suggests the clusters are actually in the data. however, it would be very good if the authors could also use an orthogonal dimensionality reduction technique, eg diffusion maps or UMAPS, to verify if they get clusters and if these clusters are compatible with those emerging with a t-SNE based visualization.

Stability: Our t-SNE begins with coordinates from a principal component analysis so as to coax similar results from different runs (now mentioned explicitly in text on line 123), but one must remember -we wrote in the discussion that the non-determinism is a weakness.

As an orthogonal dimensionality reduction method, we choose PCA to thematically combine it with the PCA analysis that addresses the next comment, and to address comments of reviewer #2 on the use and interpretation of t-SNE. The new figure S3 Fig confirms noted vector space organization trends, although the class separation is weakest here. The relevant text begins at line 330.

Initial tests with UMAP did produce a similar category / class separation to t-SNE, but in clumped local clusters. This is typical for UMAP, which is another reason why we do not present the results here. Exhaustive parameter optimization for optimal display might produce good results, but the evaluation of UMAP remains a topic for the future.

2. The embedding obtained by the authors is very intriguing, as it suggests that the lipid fingerprints lie on a low-dimensional manifold, and that the embedding extracts general features that describe the intrinsic physiochemistry of the lipids. The authors could try to use several methods to estimate the intrinsic dimensionality, say perform PCA locally and estimate how many components are necessary to retain 90% of the variance, and then take an average. Other methods exist.

Excellent suggestion – The text now states how much variance is covered by the first principal components and when 90% of the variance is reached (line 322). Additionally, we perform two forms of intrinsic dimensionality estimation related to the approach described in the comment (line 329).

Reviewer #2:

Questions/major points of revision

1. The novel approach for creating a low dimensional embedding of lipid space lies at the core of this work. However, embeddings created with tSNE can be misleading. Have the authors considered whether the 'happy accidents' of tSNE (lines 287-292) are realistic depictions of the 100-dimensional space ? See for example https://ieeexplore.ieee.org/document/9064929 (rxiv version is at https://arxiv.org/pdf/2002.06910). The authors should also consider framing their discussion in the light of Chari & Pachter's strong rebuttal regarding the use of embeddings for interpreting single cell genomics (https://journals.plos.org/ploscompbiol/article?id=10.1371/journal.pcbi.1011288). Whilst the approach here is quite distinct, much of the computational biology community are now aware of the limitations of these approaches, and clear articulation of the reasons why tsne is a good choice here is important.

We have no disagreement. We have added one of the references mentioned as well as another stating that this is no more than a visualisation method and one should not over-interpret the structure in low dimensions (paragraph starting from line 297). The newly added PCA visualizations in S3 Fig (references starting at line 330) confirm the noted trends of vector space organization but at the same time to justify the use of t-SNE to enhance the present but comparatively weak class separation.

2. The authors note in line 104 that mol2vec's approach was modified to distinguish enantiomers. Does Mol2Vec truly ignore chirality ? I was suprised at how little detail was provided regarding how mol2vec's approach was modified - it should at least warrant a reference.

The second paragraph in Methods (starting line 106) has been extended to provide more detail on how and why the modifications were done. The Mol2Vec paper does not specify the use of chirality. We inferred the ignoring of chirality from the source code, where the call to the RDKit function “AllChem.GetMorganFingerprint(mol, radius, bitInfo=info)” does not set the “useChirality” parameter (lines 99 and 151 in Mol2Vec module “features.py”), for which the default value is “False”.

3. In line 184, the authors note the embedding organises lipids with a 'general trend of more double bonds from left to right' - this could be quantitated (by pca probably), or at least visualised by summing the number of double bounds in each molecule in a sliding window across the embedding's space from left to right. Similarly, line 187's statement regarding mass distributions could be more rigorously treated if it is considered germane.

These are just qualitative observations. The wording was not too strong. We only referred to a “general trend”. The newly added S3 Fig (PCA visualization) confirms these trends.

4. A more pressing question is - are these apparent organisations of the embedding really useful for visualisation ? e.g. whilst trends in mass seem to be visualised within a class, there is no consistent orientation across all classes. It is arguable that people will gain familiarity with the landscape of the embedding (in the same way as one might learn the layout of a complex pathway map, or genome), but is there, in that case, another embedding that might even more effectively itself to that ?

No disagreement. There is no consistent orientation in 2- or 3-dimensions. We wrote in the discussion that one loses information with the dimensional reduction (paragraph starting from line 297).

5. The training data spikes do effectively demonstrate that the embedding is sensitive to differences in chemistry. What would be also interesting to measure is how sensitive the rest of the lipidome embedding is to additions - are other regions unaffected by the addition of these new species ?

The additional structures being training data, inherently influence the results but we expect only very small changes. The number of molecules added is comparatively insignificant and the classes were not changed.
The presence of biochemically uncommon hydroxylation positions in the Ceramides dataset might have an effect, which is very difficult to measure due to the inherent non-determinism of Word2Vec (and t-SNE if assessed visually). Even then, the question is, whether the effect would be detrimental given that the training data is inherently biased by the choice of classes / components made by the database curators.

6. I note a number of points of discussion regarding the Carvalho dataset:

i. Fig 4A & B show clear delineation, but is there similarly clear separation with the 2D embedding ? 3D is often impractical for publication (and generally frowned upon by dataviz experts) so it would be informative to the general reader to see the 2D equivalents - either as a figure in the main manuscript or referred to in supplementary.

The noted features regarding the hexsosyl ceramides, ether lipids (O-) and the TG / DG separation between the fat body and the lipoprotein tissues are similarly visible in the two-dimensional vector space, though everything is a bit more crowded (see embedded images below). The 3D version for the drosophila dataset was chosen for variety. To not increase the number of figures with redundant information, we will omit the 2D version.

ii. Is it possible to assign different glyphs to DG and TG rather than needing to label them ?

Currently the software only assigns distinct marker symbols to lipid categories. Marker assignment to on lipid class level will be implemented soon (but is a bit more complicated and requires the ability for the user to manually assign symbols in the interface due to a limited set of available markers in 3d scatterplots in plotly). Therefore we keep the figure as it is.

iii. Can the tool support display of the differences in lipidome between conditions in the experiments performed by Carvalho ?

It is unclear, what is meant here. The software itself is generic, and no particular feature is hard-coded. Therefore, any difference in conditions can be displayed by averaging the respective samples and filtering for the results of the averaging.

7. Embeddings limit visualisation of differences in abundance across a set of observed entities (ie lipids in this case): Figure 4C in particular demonstrates this limitation. The inset histograms in the zoomed in region (4D) are vastly superior, but this approach also takes up lots of space on the page. Have the authors considered exploring the use of small multiples to show the differences, or visualisation of signed differences using a colourscheme, overlaid on grey glyphs sized according to wild type abundances ?

This is another suggestion for the future.

8. Differential abundance analysis & Statistical rigor. It is unfortunate that the original publication does not provide the results of their own statistical analysis in a machine readable form (the lipidomeX link no longer works). This tool could thus make a very useful contribution if it were to offer such a calculation in an easy to use (and reproducible) manner. The visualisation in Figure 4E is a good start, but this visualisation is very difficult to understand without some indication of which changes are actually significant.

The software does not do proper statistics, but the suggestion of being able to import labels/colours should also go on the list for the future.

i. Line 268-269: "show that if we average the samples by genotype and challenge" - here you presumably mean 'average the samples for the same genotype in each condition' ? It's important to be clear, because the original paper demonstrates variance is negligible across replicates, so it is sufficient to compare the two genotype averages for the challenge condition.

Text changed (now at line 275).

ii. It is not clear from the manuscript that the foldchange plot is actually computed with lipid explorer. You should really make this clear! I managed to recreate the figure myself, but it might be useful to include some simple instructions on how to recreate the figure (also see point 7).

The Log2FC computation is now listed as an example operation currently performed server-side (line 160). Instructions on the usage of this and other features will be added in the interface / interactive tour / manual in the near future.

iii. Can statistical support be calculated for foldchange ? Ideally it should take into account experiment design (e.g. technical & bio-replicates) - but either way, it is important to be able to identify significance thresholds, or at the very least, provide a volcano plot.

See point 8 above.

iv. If statistical support can not be included, is there perhaps a way to import the results of an analysis performed elsewhere (here, data import/api documentation will help!)

No argument. See point 8 above.

9. Reproducibility. The authors provide all the scripts necessary to create/recreate example data sets as supplementary, and also stand-alone HTML documents produced by the tool for each figure in the paper. However, whilst the export options that the tool provides are effective, and certainly necessary, I could not see a way to save the session so it could be restored - this capability is essential for a tool that will be used to create figures for papers.

i. This limitation should be explicitly noted if it cannot be addressed in reasonable time.

This was a very instructive suggestion and a session download and upload feature has been implemented.

10. Acknowledge limitations of the visualisation capabilities of the tool. The tool appears to only allows quantitative data (abundances, fold-change, etc) to be encoded as colour, or glyph size when visualising sets of lipidomes. Many of the figures demonstrate that occlusion occurs when lipidomes are shown as circular glyphs, and additionally encoding quantities as glyph size only makes the occlusion problem worse.

No disagreement. Text added to Discussion (from line 370).

i. Ideally, visualisations of these data should be properly evaluated by a user study to ascertain whether it is actually effective for people from a variety of backgrounds. I am certain that the scaled glyph approach is far from ideal, but the nature of the embedding may lend itself to other approaches. Complex or difficult to perceive colourschemes should also be avoided. At the very least, the authors could include a few sentences describing how visualisations might be improved in the future.

See above.

ii. If possible, explore how the differences shown in 4F could be represented in a more effective way. A better approach might be to use the wild-type as a 'background' - shown in grey, and then simply highlight the difference with a single colour, rather than using two sets of coloured glyphs. Alternatively, employ a glyph that can encode two different quantities without occupying varying amounts of space: piecharts are often used for this, but other shape based approaches exist.

iii. If P-values were available, then complexity could also be reduced by simply applying a threshold to exclude insignificant differences.

These are both fine suggestions, but not for this release. Our experiments with alternative backgrounds and foregrounds until now was that we could tailor the method for specific problems, but did damage to the more common cases. The lipidome figures were updated to use grey for wild-type samples.

Minor

M1. The authors need to be more clear in their abstract and introduction that they evaluate the effectiveness of their approach using existing published datasets, and examine whether their approach allows recapitulation of the conclusions reached by the authors.

The abstract is lightly changed. We discuss the conclusions of the authors as much as we want to in the text describing figure 4.

M2. line 111. The authors note each fragment derived from the additional descriptors employed in the mol2vec approach were used to create 'separate training sentence'. Has this approach employed by others and validated rigorously ?

It has not been validated rigorously. It is the same problem as in most neural networks. One has to make some implementation decisions about the encoding. In our case, the use of a large number of radii necessitates, that we avoid creating one fragment sentence in which identifiers for very large and very small fragments are placed next to each other (which would happen at every point at which the next central atom of a set of fragments is reached). This has now been briefly described in the text (line 114). The approach is still valid for our purposes. For each radius size, the fragment vectors are trained by predicting their surrounding fragments of the same size, leading to similar fragments having similar vectors.

M3. line 182 - you should mention that figures 2C-2F are the zoomed in region of figure 2A (presumably indicated by the orange border in those plots). I would also suggest avoiding using a colour for the border that is also employed in a scale (e.g. in 1F). -- Figure 1F's mass scale should employ just a single colour, where saturation encodes mass – since the one chosen is more suited to signed quantitites such as temperature.

The style of the region borders has been adjusted to make the zooming-in more noticeable. The plots with the numerical features were adjusted and now use a single colour and saturation as the colorscale.

M4. line 193/196 - Better to swap panels for Figure 3 so the chain length/double bond spike set are labelled 3A, if they are best described first rather than second.

Done.

M5. line 230: "A user, however, would be interested in what one sees in their lipidome" – perhaps better to say '..what can be seen in their..'

M6. line 263: 'occurring' - should be 'occur'

Both changes adopted. (Now lines 236 and 270)

Notes and suggested improvements to the lipid projector tool

F1. Documentation.

i. the github repository's readme provides a very brief summary of how to get going, and cites external data employed in the development of the tool. It also cites papers with example datasets - stating that they were used in the paper. Here the authors should also make it clear that these example datasets are also included in the github repository.

The readme file has been updated accordingly.

ii. the authors include in the lipidome_projector/scripts/vector_space/README.md details of how the embedding was constructured. However, they should also either include the data used to create the accommpanying embedding, or at least a link and checksum for the data that was used and where it was retrieved. Presumably it was https://www.lipidmaps.org/files/?file=LMSD&ext=sdf.zip ?

Links to the current versions of the databases have been added to the readme file. The SwissLipids database appears to not have changed since the original access. We could not find archived versions of the LMSD to link to the originally accessed version, but all workflows work equally with the current version.

iii. The 'load data' tour was very effective as an onboarding experience. However, I would also suggest providing some brief text based documentation in the git repository regarding the expected format of data files that can be imported - for instance, one might wish to start the tool with a set of data located on the same machine, but more likely, someone is preparing data via a notebook environment.

The readme file has been updated accordingly.

The readme file has been updated with a description of the expected file formats.F2. In the Graph settings panel: does linear vs min-max scaling have any real use ? They appear to change the size of the glyphs, but visually there's not much impact (this relates to my point above regarding visual encodings: size of glyph is far less effective than position).

Different user datasets may have different abundance scale ranges, which may be suitable to different abundance-to-marker-size scaling options, so it is best to keep a variety of simple established options.

F3. Suggest default download be SVG (best for publication) - or provide a couple of options in the popup for a diagram.

SVG is implemented.

F4. Consider ways that visual complexity can be reduced:

* Use of 3D is clearly necessary when viewing both differential abundance & diversity changes, particularly since t-sne 2D plots are obviously congested, however, there is a serious visual overload issue with the default encodings. Plotly.js is a very powerful multidimensional visualisation platform, and the authors have built a very sophisticated package on top of it, but the fundamental barrier remains the complexity of lipidome data - which with this tool, often yields rather interesting cubists figures instead of intuitively understandable diagrams.

* The complexity of the form/card style user interface for creating groupings, assigning colours, doesn't really lend itself to navigating the data, since selection is primarily via the grid - which works if someone knows what they are looking for but not otherwise: It would be great if the Key allowed interactive selection of groups/subsets - allowing them to be navigated to in the table, and enable/disable their display in the plot, which would really help simplify visualisations.

F5. From a chemists perspective, representative structure is really important. Would it be possible to add sticky labels with the representative structure for a progression (as is kind of shown in the paper?). It would also be useful to be able to visualise a fleet of structures in the selected region, annotated with abundances/differences across conditions.

F6. For comparisons, small multiples might be useful to complement the quantitative visualisations of compositional statistical changes.

F4, F5 and F6 are good suggestions for future releases.

F7. As already noted earlier - the app itself appears to have no 'Save Session' function - this is essential for any data analysis tool, particularly one that also enables figures or other visualisations to be generated - since it is critical to be able to return to the data used to generate the figure.

Done.

Reviewer 3

The authors present on one hand an embedding of lipids through unsupervised machine learning and on the other hand present a visualisation tool Lipidome Projector for the resulting embedded structures.

**Problem definition:** It is clearly defined which specific problem in the field of lipidomic analysis is to be solved by the program. The program contributes to the solution of an existing problem.

**Methodology:** The choice of ML model is clearly described and justified. The data set has been sufficiently described. It is sufficiently large and diverse to provide generalizable results. The training and validation process was well documented.

**Interpretation of the results:** The results were interpreted in a biological context. The program supports the acquisition of knowledge in lipidomics and facilitates data analysis. It is described how the program can be practically applied in lipidomic research (in the analysis of complex lipid patterns).

Potential limitations were also discussed. A more fundamental problem is ambiguities in lipid notation, as well as ambiguities regarding the position, number and exact location of double bonds and hydroxylations in sphingolipids. Another problem is that a user lipidome may contain species that are not included in the training dataset (SwissLipids + LMSD).

**Reproducibility and availability:** The results and methods have been documented in such a way that other researchers can reproduce them. The code is publicly available.

**Contribution:** Lipidome Projector makes it easier to group and classify structurally similar lipids. This enables more efficient identification of patterns and similarities in lipid structure that are more difficult to detect using traditional analytical methods. Similar structures often indicate similar biochemical functions. The program makes it possible to identify functional similarities between lipids more quickly, which supports the study of lipid networks in cells.

**Similar tools:** LipidMatch is another tool that enables comprehensive identification and visualization of lipids based on mass spectrometry data. Although it is mainly used for lipid identification, it also supports the classification of lipids and the visualization of their structural relationships.LIPID MAPS, which provides detailed 2D and 3D structural information on lipids and includes visualization tools for exploring lipid metabolic pathways and interactions. It classifies lipids into major categories and shows their structure-function relationships.

We thank the reviewer for the insightful assessment of our manuscript.

Minor revisions

Supporting information „supp-1.csv“ requires a title and a short description what information the table holds for quicker understanding

A readme file accompanying this table has been added.

It could be important to check for a bias in the substructures available in the databases – due to either underlying biological patterns, differeing detectability in the LC-MS or simply through overrepresentation by resarch interest (e.g. focus on specific diseases) and to add a potential bias into the discussion how generalizable Lipidome Projector could be for edge cases.

Two sentences (starting at line 306) have been added to acknowledge bias in the databases used for training.

The reproducablility of Lipidome Projector could be improved significantlly if the package verions could either be added in the script, or in the pyproject.toml

Semantic versioning is present in the pyproject.toml.

There are two sentences that are a bit misunderstandable:

Page 3 – Line 46: „but a list of 10²-104 lipids and quantities “could be addressed as „lipids and lipid quantities“ or „lipids and their quantities“

Page 13 – Line 286-289: „…it is remarkable that moving a hydroxylation along a chain gives a set of points near each other and almost lying on a smooth curve.“ This does not sound grammatically correct.

Both fixed.
